# Investigating the role of Kinesin family in lung adenocarcinoma via integrated bioinformatics approach

**DOI:** 10.1038/s41598-023-36842-6

**Published:** 2023-06-17

**Authors:** Gulnaz Tabassum, Prithvi Singh, Rishabh Gurung, Mohammed Ageeli Hakami, Nada Alkhorayef, Ahad Amer Alsaiari, Leena S. Alqahtani, Mohammad Raghibul Hasan, Summya Rashid, Atul Kumar, Kapil Dev, Ravins Dohare

**Affiliations:** 1 Department of Biotechnology, Faculty of Natural Sciences, Jamia Millia Islamia, New Delhi, 110025 India; 2 Centre for Interdisciplinary Research in Basic Sciences, Jamia Millia Islamia, New Delhi, 110025 India; 3 Department of Clinical Laboratory Sciences, College of Applied Medical Sciences, Al- Quwayiyah, Shaqra University, Riyadh, 13343 Saudi Arabia; 4 Department of Clinical Laboratory Sciences, College of Applied Medical Sciences, Taif University, Taif, 21944 Saudi Arabia; 5 Department of Biochemistry, College of Science, University of Jeddah, Jeddah, 23445 Saudi Arabia; 6 Department of Pharmacology and Toxicology, College of Pharmacy, Prince Sattam Bin Abdulaziz University, Alkharj, 16278 Saudi Arabia

**Keywords:** Computational biology and bioinformatics, Genetics, Immunology, Structural biology, Systems biology

## Abstract

Lung cancer is the leading cause of mortality from cancer worldwide. Lung adenocarcinoma (LUAD) is a type of non-small cell lung cancer (NSCLC) with highest prevalence. Kinesins a class of motor proteins are shown to be involved in carcinogenesis. We conducted expression, stage plot and survival analyses on kinesin superfamily (KIF) and scrutinized the key prognostic kinesins. Genomic alterations of these kinesins were studied thereafter via cBioPortal. A protein–protein interaction network (PPIN) of selected kinesins and 50 closest altering genes was constructed followed by gene ontology (GO) term and pathway enrichment analyses. Multivariate survival analysis based on CpG methylation of selected kinesins was performed. Lastly, we conducted tumor immune infiltration analysis. Our results found *KIF11*/*15*/*18B*/*20A*/*2C*/*4A*/*C1* to be significantly upregulated and correlated with poor survival in LUAD patients. These genes also showed to be highly associated with cell cycle. Out of our seven selected kinesins, *KIFC1* showed the highest genomic alteration with highest number of CpG methylation. Also, CpG island (CGI) cg24827036 was discovered to be linked to LUAD prognosis. Therefore, we deduced that reducing the expression of *KIFC1* could be a feasible treatment strategy and that it can be a wonderful individual prognostic biomarker. CGI cg24827036 can also be used as a therapy site in addition to being a great prognostic biomarker.

## Introduction

Lung cancer (LC) is a prevalent and deadly disease that ranks first among cancers in terms of death and 2nd most diagnosed cancer in both genders globally^1^. The etiological and molecular heterogeneity of LC contributes greatly to treatment failure and adverse survival outcomes^2,3^. Most LCs diagnosed are malignant epithelial tumours, which can be further classified as small-cell lung carcinoma (SCLC) or non-small cell lung carcinoma (NSCLC). NSCLC accounts for 85–90% of lung malignancies, with lung adenocarcinoma (LUAD) and lung squamous cell carcinoma (LUSC) being the most frequent subtypes^4,5^. LUAD and LUSC can be classified into four stages, referred to as $\mathrm{I}$, $\mathrm{II}$, $\mathrm{III}$, and $\mathrm{IV}$, as per the tumor node metastasis (TNM) taxonomy^6^. The early, non-metastatic stage is referred to as stage $\mathrm{I}$. Stages $\mathrm{II}$ and $\mathrm{III}$ typically represent the intermediate, regional lymphatic metastatic phases, with stage $\mathrm{III}$ exhibiting more significant metastasis in the lymphatic region than stage $\mathrm{II}$. Meanwhile, stage $\mathrm{IV}$ often denotes a late stage with distant metastases^6^.

Despite evidence that smoking increases the risk of LUAD, it is currently the most common subgroup of LC among non-smokers and women^7,8^. Patients with LUAD typically have a poor prognosis and frequently show local progression or metastasis when diagnosed^9^. However, LUSC is more prevalent in men than in women and has been strongly linked to smoking^10^. Although chemotherapy, radiation, and targeted medicines are widely employed, therapeutic resistance to these treatments is a primary cause of treatment failure. Understanding the underlying molecular pathways of carcinogenesis is thus critical for developing effective LC therapies.

Human kinesin superfamily members (KIFs) consist of $14$ kinesin family members, kinesin-1 to kinesin-14, according to the standardized nomenclature adopted by the kinesin research group^11^. There are $45$ members in the KIFs superfamily, including $39$ N-kinesins, three M-kinesins, and three C-kinesins^12^. KIF proteins are a family of motor proteins that move molecules and depend on microtubules. They have ATPase activity as well as motion characteristics. They bind to microtubules and then move along the microtubules, carrying protein complexes, organelles, and messenger RNAs (mRNAs)^12–14^. In recent years, it has come to light that several KIFs contribute uniquely to the process of mitosis, also known as cell division, by taking part in the motion of chromosomes and spindles^15,16^. Additionally, individual kinesins are also essential for a number of other cellular processes, such as endocytosis and transcytosis, intracellular transport^14^.

Mitosis, the process by which eukaryotic cells divide, creates two daughter cells with approximately equal amounts of the cell's nucleus, cytoplasm, organelles, and membrane. It is possible that mistakes in this process could lead to the death of cell, abnormalities (including deletion of gene, translocation of chromosome, or the duplication of chromosomes), and even cancer. Since mitosis is so intricately controlled therefore, any alteration or changes in KIF expression or function could potentially cause cancer. Kinesins and motor proteins with abnormal expression are crucial mitotic process regulators and potential targets in human malignancies^17–19^. Human cancer is a genetic disorder characterized by uncontrolled cell development, hence inhibiting kinesins may provide a unique approach to managing this disease.

Thus, identifying anomalous kinesin gene expression could be utilized as a biomarker for early tumor diagnosis and targeting kinesins could also be a novel approach for cancer therapy. Therefore, in the current study, we conducted a comprehensive bioinformatics analysis to identify the key kinesins influencing the prognosis of LUAD cancer patients. We performed expression and stage plot analyses of KIFs across the cancer genome atlas (TCGA)-LUAD patient samples and reported only significant ones. Next, we proceeded with overall survival (OS) analysis followed by mutational, enrichment, and protein–protein interaction network (PPIN) analyses. At last, we obtained *KIFC1* as final prognostic biomarker responsible for LUAD pathogenesis. *KIFC1* can be further used for early detection of LUAD patients and targeted therapy or personalized medicine.

## Materials and methods

### Kinesins expression and stage analysis across LUAD cohort

Gene expression profiling interactive analysis v2 (GEPIA 2) web-based tool^20^ (http://gepia2.cancer-pku.cn/) was accessed for comparing the relative mRNA expression level of all kinesin family members across TCGA-LUAD cohort and matched TCGA normal and GTEx data. The expression values from GEPIA were already transformed into ${\mathrm{log}}_{2}(\mathrm{TPM}+1)$ values followed by differential analysis. Pathological stage plot analysis was also done with GEPIA 2 to investigate the kinesin family members' expression with respect to different pathological stages in LUAD. The threshold used in GEPIA for mRNA expression level comparison across LUAD and normal samples were as follows: $p \mathrm{value}<0.05$ and $\left|{\mathrm{log}}_{2}(\mathrm{fold change})\right|>1$. Kinesins statistically significant in both expression and stage plot analyses were selected for further analyses.

### Prognostic analysis of kinesins across LUAD cohort

Kaplan–Meier (KM) plotter^21,22^ (https://kmplot.com/analysis/) was queried for prognostic analysis of kinesins having significance in expression and stage plot analyses. We generated KM plots of only those kinesins which showed significant OS across LUAD patient samples. The microarray LUAD patients were bifurcated into higher and lower expression groups based on their median values. The redundant samples were removed in the quality control section, and biased arrays were excluded. Hazard ratio (HR) with the corresponding 95% confidence interval (CI), ${\text{logrank}}\;p\;{\text{value}}$ and median survival were calculated. ${\text{logrank}}\;p\;{\text{value}} < 0.05$ was considered as a statistically significant threshold for assessing the prognosis of kinesins between two expression groups.

### Validation of prognostic kinesins using cBioPortal

We queried the cBioPortal for Cancer Genomics^23^ (https://www.cbioportal.org/) for investigating the mutations and putative copy number alterations (CNAs) of prognostically significant kinesins. The LUAD dataset (TCGA, Firehose Legacy) was chosen to perform our analysis.

### Validation of prognostic kinesins using GEO and correlation analysis

We queried the NCBI- GEO^24^ (https://www.ncbi.nlm.nih.gov/geo/) using “LUAD” and “Lung Adenocarcinoma” as suitable keywords for extracting LUAD-associated mRNA expression profile. All the search results were further trimmed down in accordance with the following inclusion criteria: (1) the samples present in dataset(s) must belong to ‘Homo Sapiens’; (2) dataset(s) type must be ‘expression profiling by array’; (3) both preprocessed and raw files of the dataset(s) must be available; (4) the dataset(s) submission date to GEO must be within last $10$ years (i.e. 2012–2022); (5) the dataset(s) must be comprising both tumor and healthy control tissue samples; (6) the dataset(s) must comprise at least $25$ samples. Any abstracts, case reports, review-based articles, cell-line-based experimental study designs, and studies devoid of healthy controls or non-human samples were excluded. Sequential steps of batch correction, probe ID to gene mapping, and duplicacy removal were performed as discussed previously^25^. The DEGs were screened corresponding to a Benjamini-Hochberg (BH)—*p* value < 0.0 and $\left| {{\text{log}}_{2} \left( {\text{fold change}} \right)} \right| > 0.5$ utilizing limma^26^. The presence of key prognostic kinesins was checked in the DEGs list. Next, we accessed GEPIA 2 to perform pairwise correlation analysis of key prognostic kinesins across TCGA-LUAD and normal patients. *p* value < 0.05 was considered as the cutoff for statistical significance.

### PPIN construction and enrichment analysis

A PPIN was constructed between the prognostically significant kinesins and top $50$ frequently altered genes corresponding to a default confidence (i.e., interaction score $>0.4$) using Search Tool for the Retrieval of Interacting Genes (STRING) v11.5 web-based tool^27^ (https://string-db.org/) and visualized via Cytoscape v3.9.1^28^. Top $10$ significant (i.e., $\mathrm{p}-\mathrm{value}<0.05$) pathway and gene ontology (GO) terms for the constructed PPIN items were compiled using Enrichr web server^29^ (https://maayanlab.cloud/Enrichr). Kyoto Encyclopedia of Genes and Genomes (KEGG)^30–32^, GO-Biological Process (BP), GO-Molecular Function (MF), and GO-Cellular Compartment (CC) libraries were used for pathway and GO terms.

### Tumor infiltration analysis

We looked into the relationship between mRNA expression levels of prognostically significant kinesins with tumor-infiltrating immune cells such as B cells, ${\mathrm{CD}8}^{+}$T cell, macrophage, and neutrophils across TCGA-LUAD patients using TIMER 2.0^33^ (http://timer.cistrome.org/). To assess the statistical significance, Spearman correlation was used.

### Methylation analysis

Prognostic analysis of single CpG methylation of selected genes of kinesin family in LUAD patients was conducted using MethSurv^34^ (https://biit.cs.ut.ee/methsurv), a web tool for multivariate survival analysis based on CpG methylation data.

## Results

### Kinesins expression and stage plot analysis across LUAD cohort

**Figure 1:**
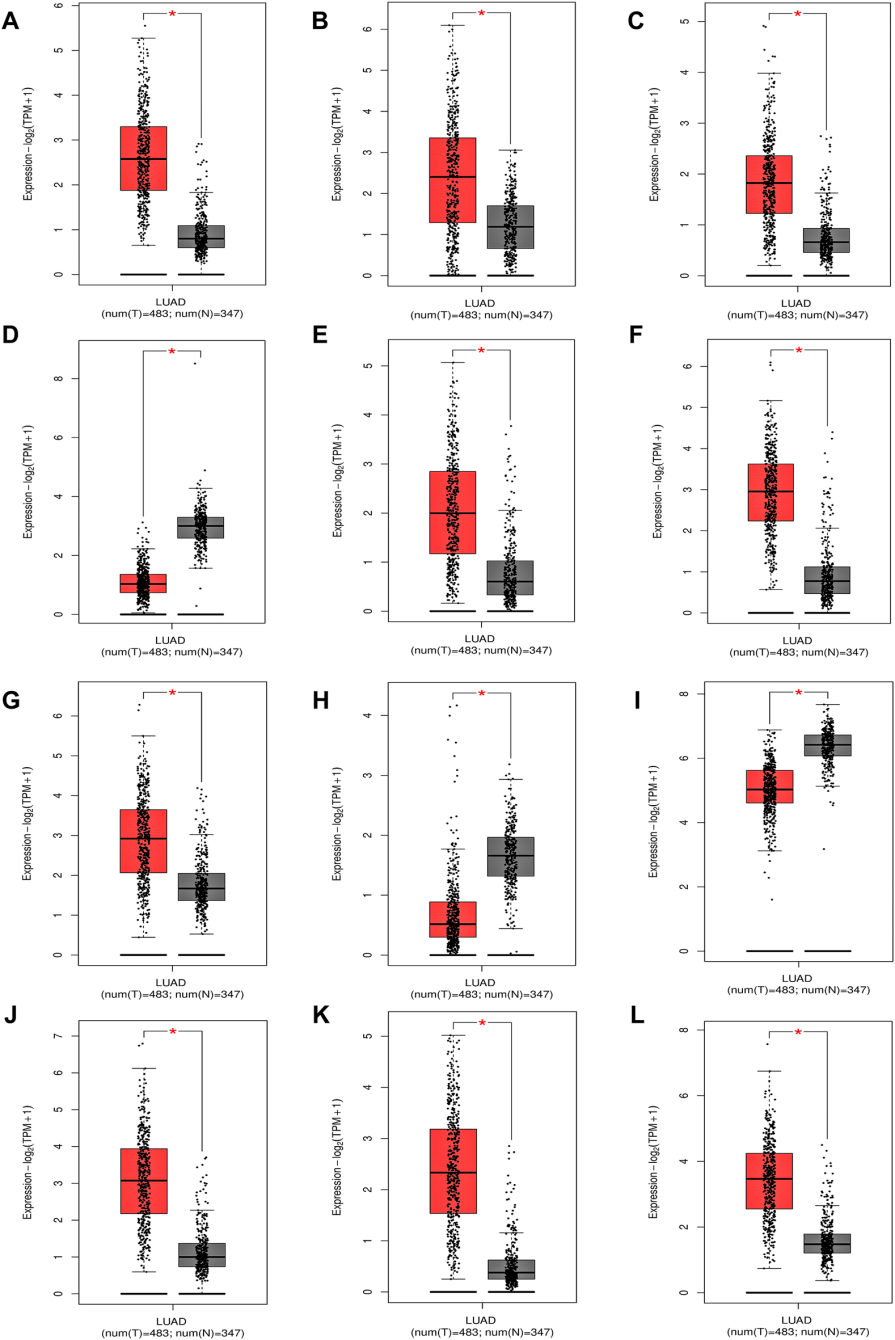
Box-and-whisker plots displaying the relative mRNA expression levels of (**A**) KIF11, (**B**) KIF12, (**C**) KIF15, (**D**) KIF17, (**E**) KIF18B, (**F**) KIF20A, (**G**) KIF23, (**H**) KIF26A, (**I**) KIF1C, (**J**) KIF2C, (**K**) KIF4A, (**L**) KIFC1 across TCGA-LUAD and normal samples. Grey-and red-colored box areas signify normal and tumor patient samples. The top and bottom of the boxes signify 75th and 25th percentile of distribution. Horizontal lines within the boxes represent the median values while minimum and maximum values label the axes endpoints. *$p\;{\text{value}} < 0.05$.

**Figure 2:**
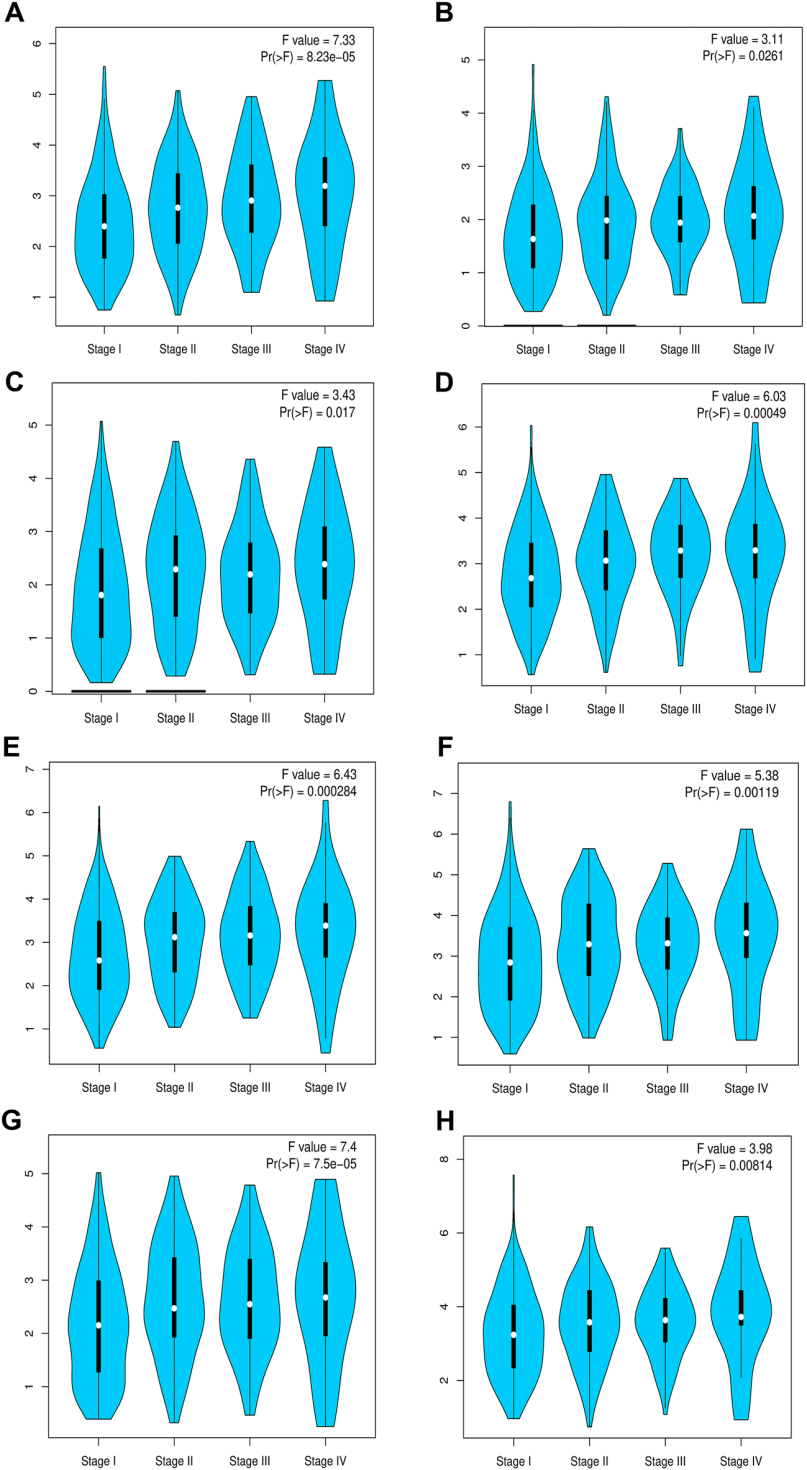
Violin plots displaying association between significant TNM sub-stages and mRNA expression levels of (**A**) KIF11 (**B**) KIF15, (**C**) KIF18B, (**D**) KIF20A, (**E**) KIF23, (**F**) KIF2C, (**G**) KIF4A, (**H**) KIFC1 across TCGA-LUAD cohort. The black-colored vertical bars and white-colored dots signify interquartile ranges and median, respectively. The ordinate and abscissa depict expression levels of these genes and various stages. Distribution density is represented by the width of turquoise-colored shapes, respectively.

All kinesins' relative mRNA expression distribution across TCGA-LUAD cohort ($483$ tumor and $347$ normal) was compiled utilizing GEPIA. *KIF11*, *KIF12*, *KIF15*, *KIF23*, *KIF18B*, *KIF20A*, *KIF2C*, *KIF4A*, *KIFC1* expression levels were significantly upregulated while *KIF17*, *KIF26A*, *KIF1C* expressions were significantly downregulated in tumor samples as shown by the box-and-whisker plots in Fig. 1A–L. All these significantly expressed kinesins were carried further to stage plot analysis. The pathological sub-stage analysis as shown by violin plots in Fig. 2A–H revealed that overexpressed levels of *KIF11*, *KIF15*, *KIF23*, *KIF18B*, *KIF20A*, *KIF2C*, *KIF4A*, *KIFC1* significantly correlated with advanced TNM stages across TCGA-LUAD cohort.

### Prognostic analysis of kinesins across LUAD cohort

**Figure 3:**
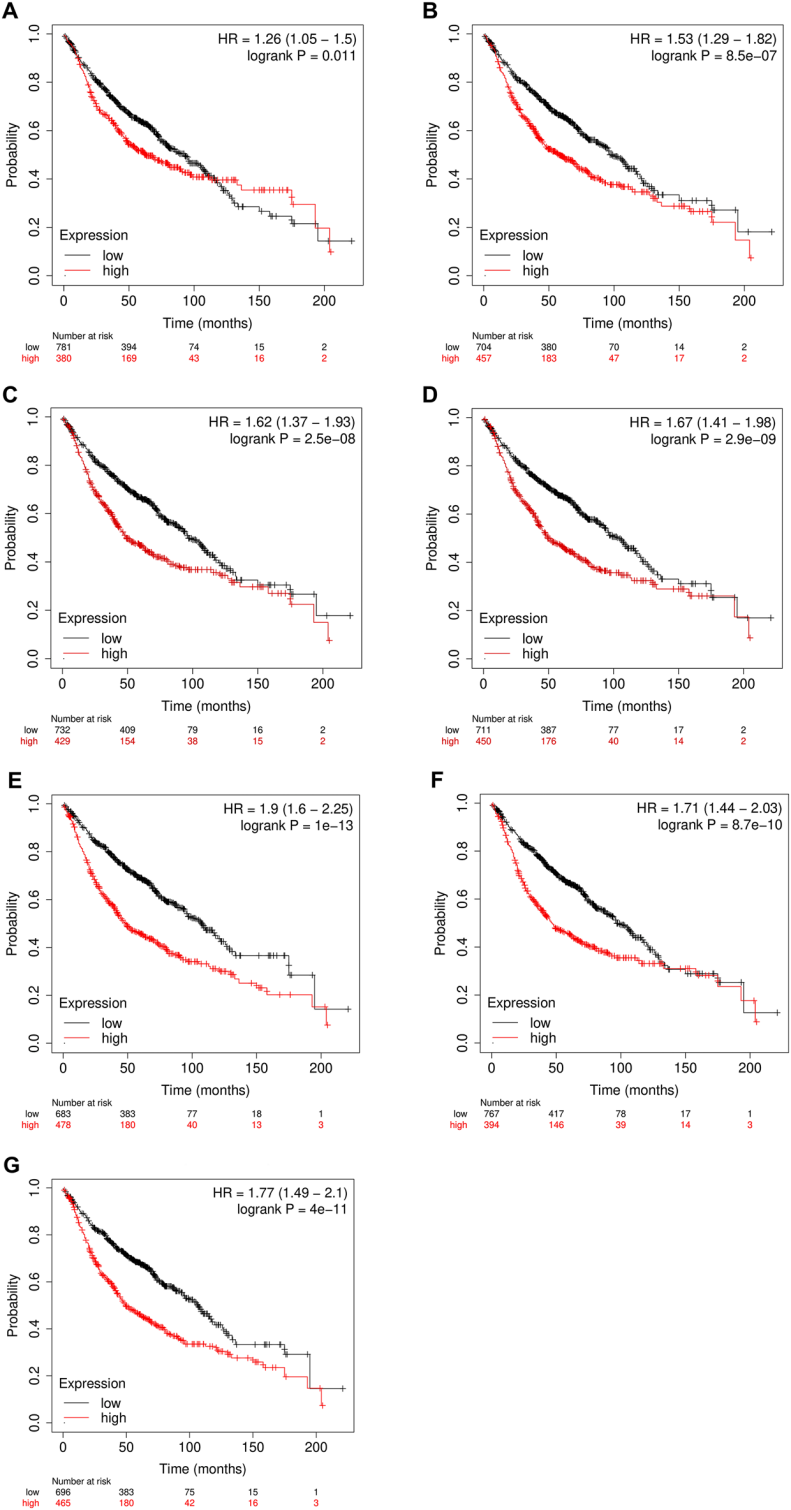
KM plots showing the OS of (**A**) KIF11 (**B**) KIF15, (**C**) KIF18B, (**D**) KIF20A, (**E**) KIF2C, (**F**) KIF4A, (**G**) KIFC1 across LUAD microarray cohort. Red and black colors signify higher and lower expression groups.

Using KM plotter, prognostic analysis was performed on *KIF11*, *KIF15*, *KIF23*, *KIF18B*, *KIF20A*, *KIF2C*, *KIF4A*, *KIFC1* to determine the correlation between their mRNA expression levels and risk of $513$ LUAD patient samples. The KM plots as shown in Fig. 3A–G revealed significantly poor OS of LUAD patients when mRNA expression levels of *KIF11*, *KIF15*, *KIF18B*, *KIF20A*, *KIF2C*, *KIF4A*, and *KIFC1* were high. The low and high expression cohort median survival time, HR, $95\mathrm{\% CI}$, and $\mathrm{logrank p}-\mathrm{value}$ of each kinesin is detailed in Supplementary Table S1, respectively.

### Validation of key prognostic kinesins using cBioPortal

**Figure 4:**
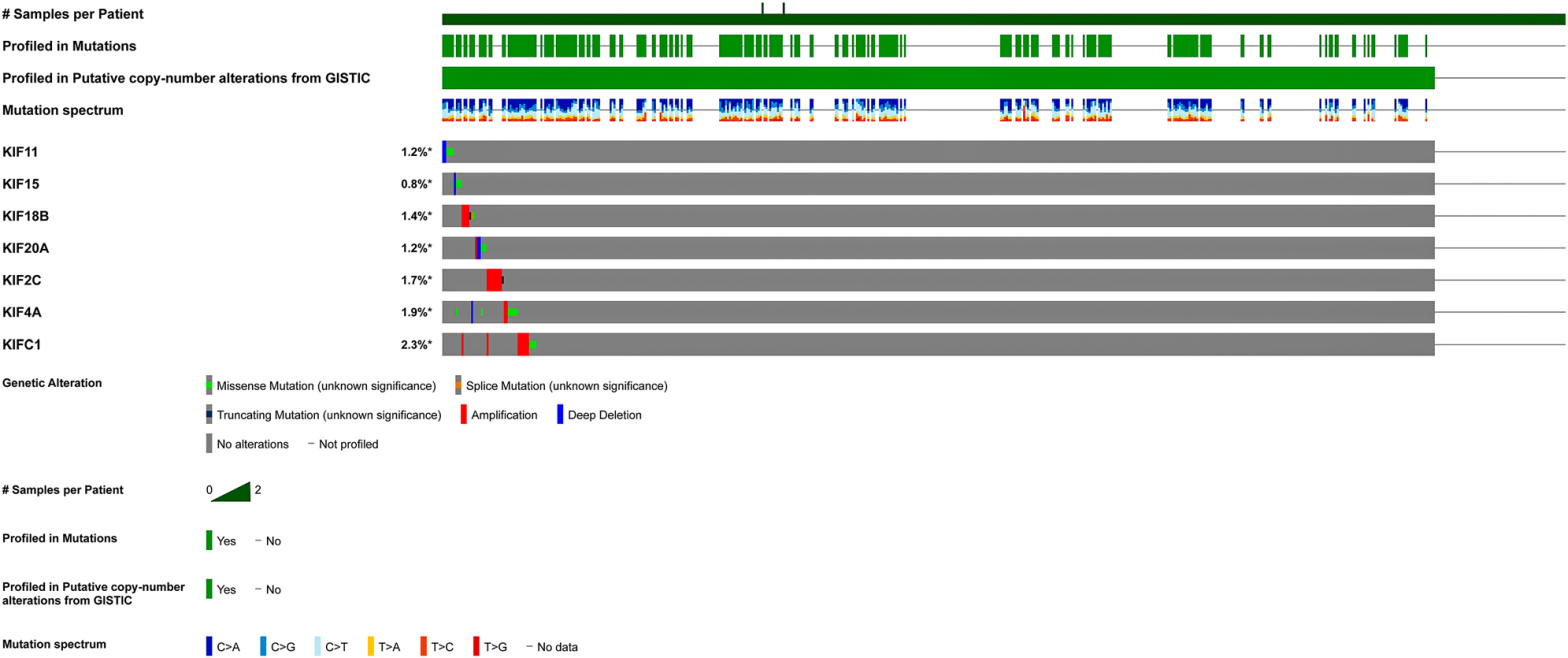
OncoPrint summarizing genomic alterations of key prognostic kinesins across TCGA-LUAD cohort comprising 584 patient samples. The bottom row represents frequency of genomic alterations in KIF11, KIF15, KIF18B, KIF20A, KIF2C, KIF4A, KIFC1 with red, blue, green, orange, and grey bars signifying amplifications, deep deletions, missense, splice, and truncating mutations, respectively. First, second, third, fourth, and fifth rows depicts the clinical annotation bars such as profiled in putative copy-number alterations from GISTIC, mutation spectrum, sex, tissue source site, and mutation count, respectively.

We used cBioPortal to validate the specific genetic modifications associated with key prognostic kinesins (i.e., *KIF11*, *KIF15*, *KIF18B*, *KIF20A*, *KIF2C*, *KIF4A*, *KIFC1*) across LUAD dataset (TCGA, Firehose legacy) comprising $584$ tumor patient samples. OncoPrint results for these queried genes as represented in Fig. 4 revealed genetic alterations in $8\mathrm{\%}$ ($49/584$) patient samples. As observed, *KIFC1* showed maximum mutation frequency ($2.3\mathrm{\%}$) as compared to others. The cancer type summary analysis revealed the overall alteration frequency of these genes as shown in Supplementary Figure S1. We observed $0.78\mathrm{\%}$ ($4/516$ cases) missense mutation and $0.39\mathrm{\%}$ ($2/516$ cases) deep deletion in case of *KIF11*. In case of *KIF15*, we observed $0.58\mathrm{\%}$ ($3/516$ cases) missense mutation and $0.19\mathrm{\%}$ ($1/516$ case) deep deletion. In case of *KIF18B*, we observed $0.78\mathrm{\%}$ ($4/516$ cases) amplification and $0.58\mathrm{\%}$ ($3/516$ cases) missense mutation. In case of *KIF20A*, we observed $0.58\mathrm{\%}$ (3/516 cases) missense mutation, 0.39% (2/516 cases) deep deletion, and 0.19% (1/516 case) amplification. In case of *KIF2C*, we observed 0.19% (1/516 case) truncating mutation and 1.55% (8/516 cases) amplification. In case of *KIF4A*, we observed 0.19% (1/516 case) deep deletion, 0.39% (2/516 cases) amplification, and 1.36% (7/516 cases) missense mutation. In case of *KIFC1*, we observed 1.55% (8/516 cases) amplification and 0.78% (4/516 cases) missense mutation.

### Validation using GEO and correlation analysis

As per the specified inclusion and exclusion criteria we chose GSE43458 (30 healthy control + 80 tumor tissues) and GSE116959 (11 healthy control + 57 tumor tissues) LUAD-associated mRNA expression profiles. A total of 2861 and 5128 DEGs were screened corresponding to GSE43458 and GSE116959 as per the specified threshold. The lists of DEGs are shown in Supplementary Tables S2 and S3. All the key prognostic kinesins (i.e., *KIF11*, *KIF15*, *KIF18B*, *KIF20A*, *KIF2C*, *KIF4A*, and *KIFC1*) were present in the DEGs lists of both datasets, thus confirming their validation in external GEO datasets. Strikingly, all the prognostic kinesins were upregulated among DEGs list and matched with the primary results obtained form GEPIA 2. Scatterplots showing pairwise correlations among these key prognostic kinesins are demonstrated in Supplementary Figures S2–S5. Significantly highest correlation between *KIF4A* and *KIF2C* (${\text{R}} = 0.95$, $p\;{\text{value}} = 6.9 \times 10^{ - 269}$) was observed.

### PPIN construction and enrichment analysis

**Figure 5:**
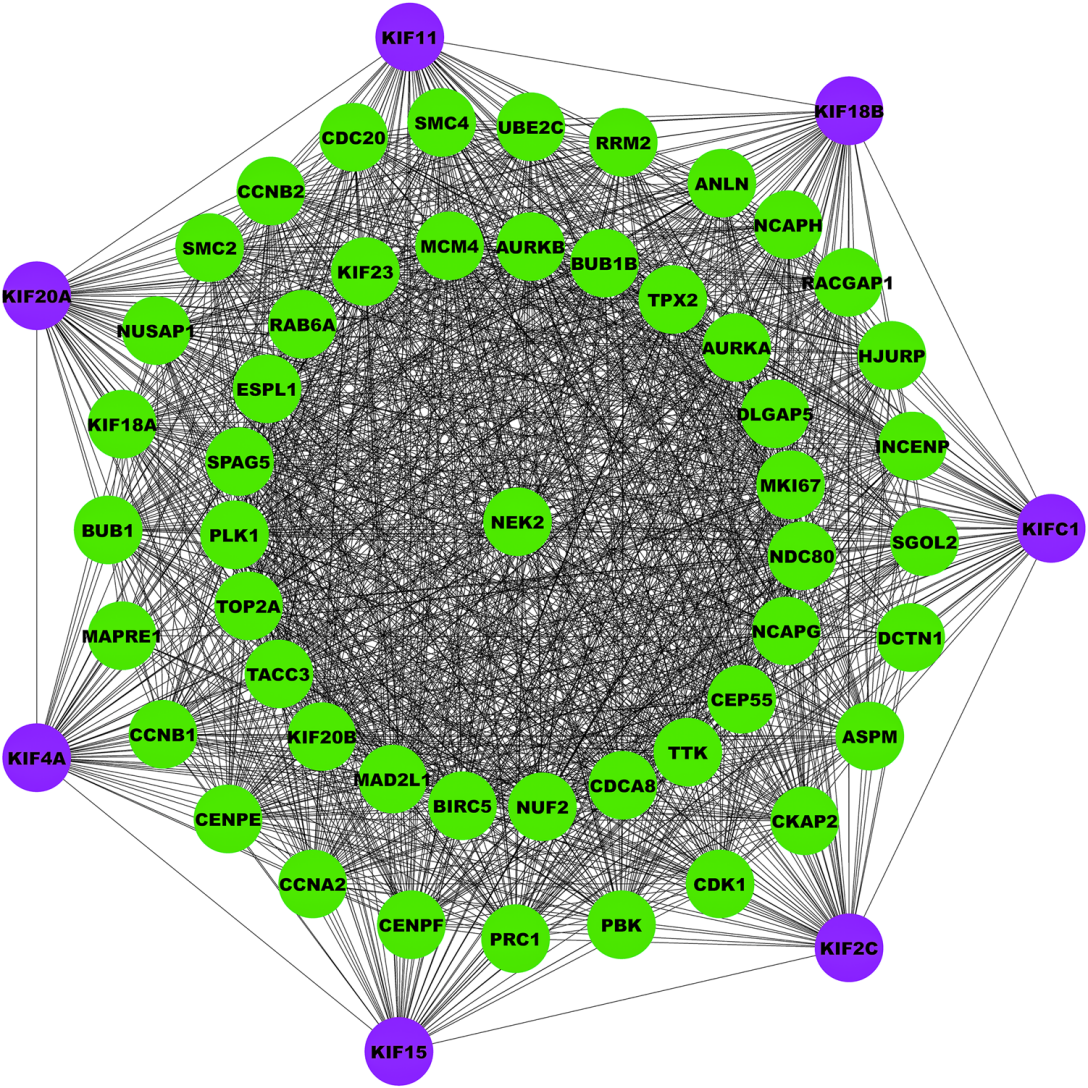
PPIN comprising 57 nodes and 1455 edges. Magenta-colored nodes represent prognostic kinesins and green-colored nodes represent top 50 frequently altered genes.

**Figure 6:**
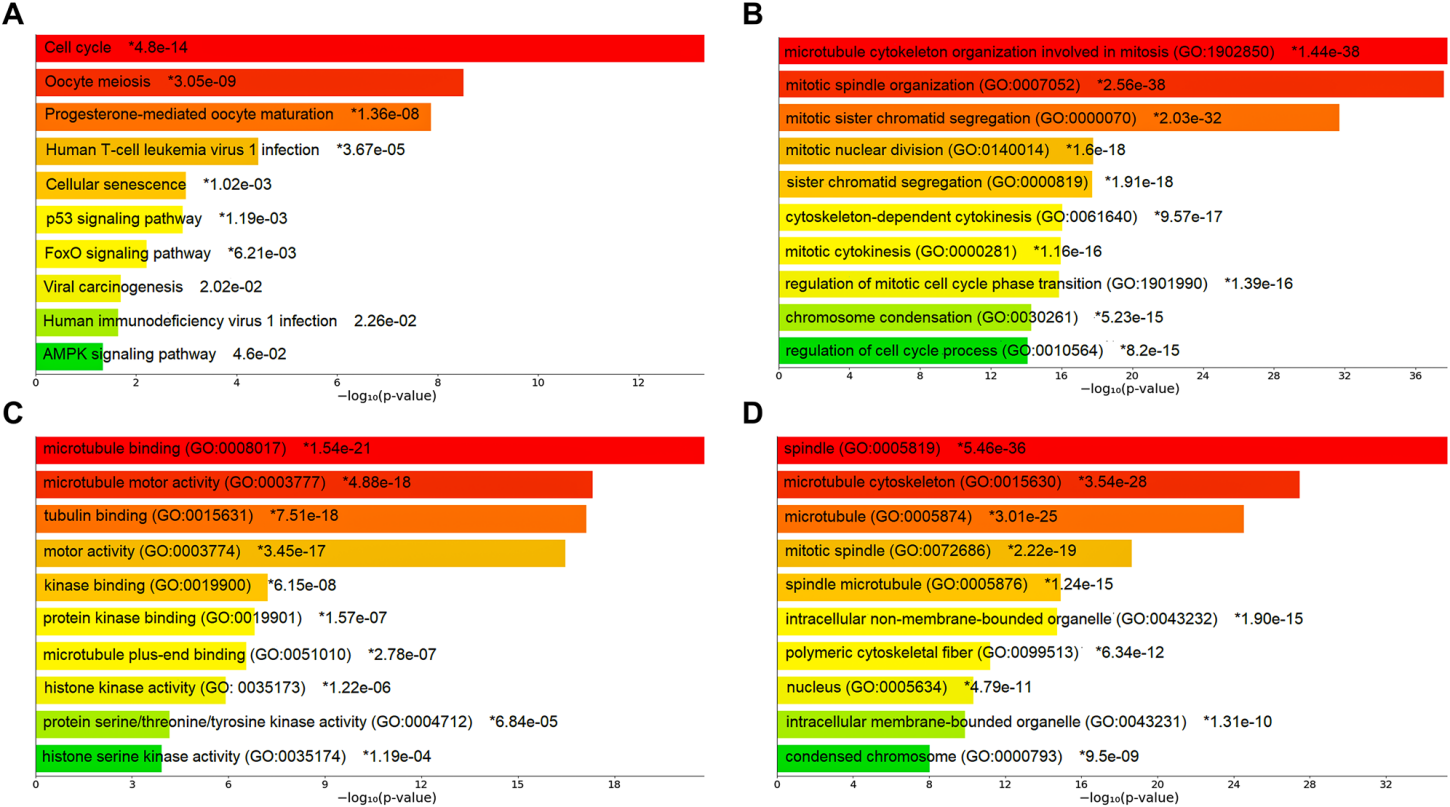
Barplots showing top 10 significantly enriched (**A**) pathways, (**B**) GO-BP, (**C**) GO-MF, (**D**) GO-CC terms with respect to *p* values. The color of bars varies in accordance with *p* values with red signifying lowest *p* values and green signifying highest *p* values. Asterisk signs represent the terms are also significant according to FDR.

Our PPIN comprised a total of 57 nodes and 1455 edges as shown in Fig. 5. Within PPIN, degree, betweenness, and closeness values ranged from 4 to 56, 0.07 to 40.44, and 0.51 to 1. The average degree, betweenness, and closeness of PPIN were 51.05, 4.94, and 0.931. Topological/centrality measures like node degree, betweenness, closeness, clustering coefficient, neighborhood connectivity, and average shortest path length of PPIN are demonstrated in Supplementary Figure S6. Subsequently, we performed pathway and GO term enrichment analysis on key prognostic kinesins and associated top 50 frequently altered genes. Barplots showing top 10 significantly enriched pathway and GO terms is shown in Fig. 6. The most significant pathway, GO-BP, GO-MF, GO-CC terms were cell cycle ($p\;{\text{value}} = 4.8 \times 10^{ - 14}$), microtubule cytoskeleton organization involved in mitosis ($p\;{\text{value}} = 1.44 \times 10^{ - 38}$), microtubule binding ($p\;{\text{value}} = 1.54 \times 10^{ - 21}$), spindle ($p\;{\text{value}} = 5.46 \times 10^{ - 36}$). Most number of genes corresponding to pathway, GO-BP, GO-MF, GO-CC terms were 11, 25, 18, 38 for cell cycle, mitotic spindle organization, microtubule binding, intracellular membrane-bounded organelle.

### Tumor infiltration analysis

**Figure 7:**
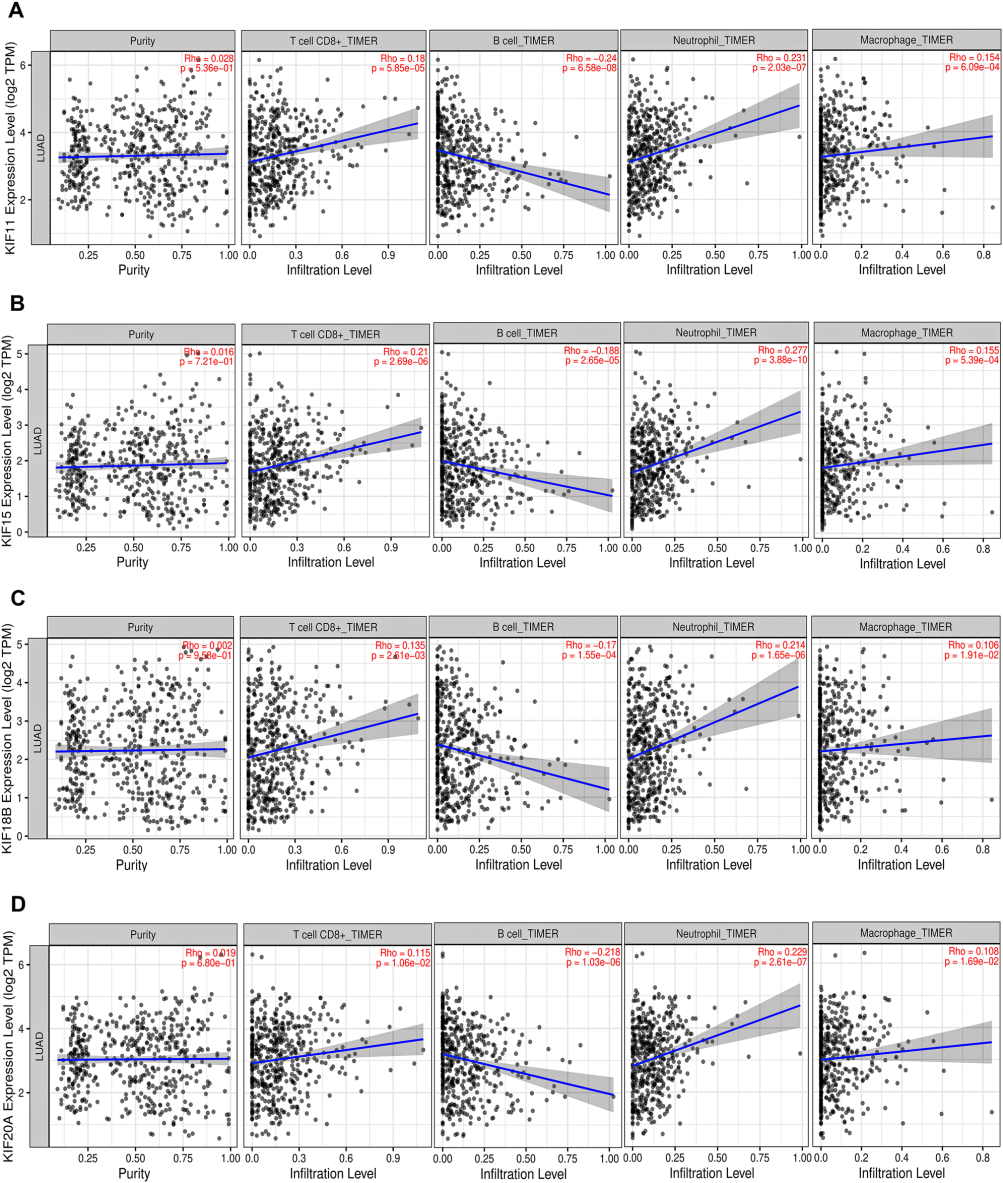

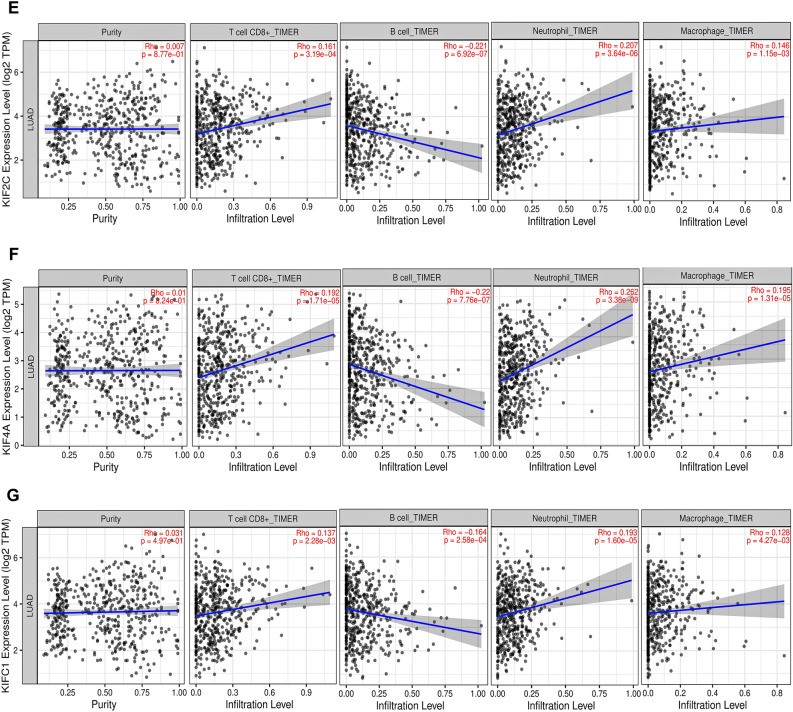
Scatterplots showing significant correlations of (**A**) KIF11, (**B**) KIF15, (**C**) KIF18B, (**D**) KIF20A, (**E**) KIF2C, (**F**) KIF4A, (**G**) KIFC1 with infiltrating levels of CD8^+^T cell, B cells, neutrophils, and macrophages across TCGA-LUAD cohort. Spearman’s correlation value and estimated statistical significance were shown as the legends for each scatter plot.

Correlation of *KIF11*, *KIF15*, *KIF18B*, *KIF20A*, *KIF2C*, *KIF4A*, *KIFC1* mRNA expression levels with tumor purity and infiltrating levels of neutrophils, macrophages, B cells, and CD8^+^T cell across TCGA-LUAD cohort are shown by scatterplots in Fig. 7. *KIF11* displayed significant positive correlations with infiltrating levels of CD8^+^T cell (${\text{r}} = 0.18$, $p = 5.85 \times 10^{ - 5}$), neutrophils (${\text{r}} = 0.231$, $p = 2.03 \times 10^{ - 7}$), and macrophages (${\text{r}} = 0.154$, $p = 6.09 \times 10^{ - 4}$). *KIF15* displayed significant positive correlations with infiltrating levels of ${\text{CD}}8^{ + }$T cell (${\text{r}} = 0.21$, $p = 2.69 \times 10^{ - 6}$), neutrophils (${\text{r}} = 0.277$, $p = 3.88 \times 10^{ - 10}$), and macrophages (${\text{r}} = 0.155$, $p = 5.39 \times 10^{ - 4}$). *KIF18B* displayed significant positive correlations with infiltrating levels of CD8^+^T cell (${\text{r}} = 0.135$, $p = 2.61 \times 10^{ - 3}$), neutrophils (${\text{r}} = 0.214$, $p = 1.65 \times 10^{ - 6}$), and macrophages (${\text{r}} = 0.106$, $p = 1.91 \times 10^{ - 2}$). *KIF20A* displayed significant positive correlations with infiltrating levels of CD8^+^T cell (${\text{r}} = 0.115$, $p = 1.06 \times 10^{ - 2}$), neutrophils (${\text{r}} = 0.229$, $p = 2.61 \times 10^{ - 7}$), and macrophages (${\text{r}} = 0.108$, $p = 1.69 \times 10^{ - 2}$). *KIF2C* displayed significant positive correlations with infiltrating levels of CD8^+^T cell (${\text{r}} = 0.161$, $p = 3.19 \times 10^{ - 4}$), neutrophils (${\text{r}} = 0.207$, $p = 3.64 \times 10^{ - 6}$), and macrophages (${\text{r}} = 0.146$, $p = 1.15 \times 10^{ - 3}$). *KIF4A* displayed significant positive correlations with infiltrating levels of ${\text{CD}}8^{ + }$T cell (${\text{r}} = 0.192$, $p = 1.71 \times 10^{ - 5}$), neutrophils (${\text{r}} = 0.262$, $p = 3.38 \times 10^{ - 9}$), and macrophages (${\text{r}} = 0.195$, $p = 1.31 \times 10^{ - 5}$). *KIFC1* displayed significant positive correlations with infiltrating levels of CD8^+^T cell (${\text{r}} = 0.137$, $p = 2.28 \times 10^{ - 3}$), neutrophils (${\text{r}} = 0.193$, $p = 1.60 \times 10^{ - 5}$), and macrophages (${\text{r}} = 0.128$, $p = 4.27 \times 10^{ - 3}$). *KIF11* (${\text{r}} = - 0.24$, $p = 6.58 \times 10^{ - 8}$), *KIF15* (${\text{r}} = - 0.188$, $p = 2.65 \times 10^{ - 5}$), *KIF18B* (${\text{r}} = - 0.17$, $p = 1.55 \times 10^{ - 4}$), *KIF20A* (${\text{r}} = - 0.218$, $p = 1.03 \times 10^{ - 6}$), *KIF2C* (${\text{r}} = - 0.221$, $p = 6.92 \times 10^{ - 7}$), *KIF4A* (${\text{r}} = - 0.22$, $p = 7.76 \times 10^{ - 7}$), *KIFC1* (${\text{r}} = - 0.164$, $p = 2.58 \times 10^{ - 4}$) showed significant negative correlations with infiltrating levels of B cells. In addition, *KIF11* (${\text{r}} = 0.028$, $p = 5.36 \times 10^{ - 1}$), *KIF15* (${\text{r}} = 0.016$, $p = 7.21 \times 10^{ - 1}$), *KIF18B* (${\text{r}} = 0.002$, $p = 9.58 \times 10^{ - 1}$), *KIF20A* (${\text{r}} = 0.019$, $p = 6.80 \times 10^{ - 1}$), *KIF2C* (${\text{r}} = 0.007$, $p = 8.77 \times 10^{ - 1}$), *KIF4A* (${\text{r}} = 0.01$, $p = 8.24 \times 10^{ - 1}$), *KIFC1* (${\text{r}} = 0.031$, $p = 4.97 \times 10^{ - 1}$) showed nonsignificant positive correlations with tumor purity across TCGA-LUAD cohort.

### Prognostic analysis based on single CpG methylation of selected kinesins in LUAD patients

**Figure 8:**
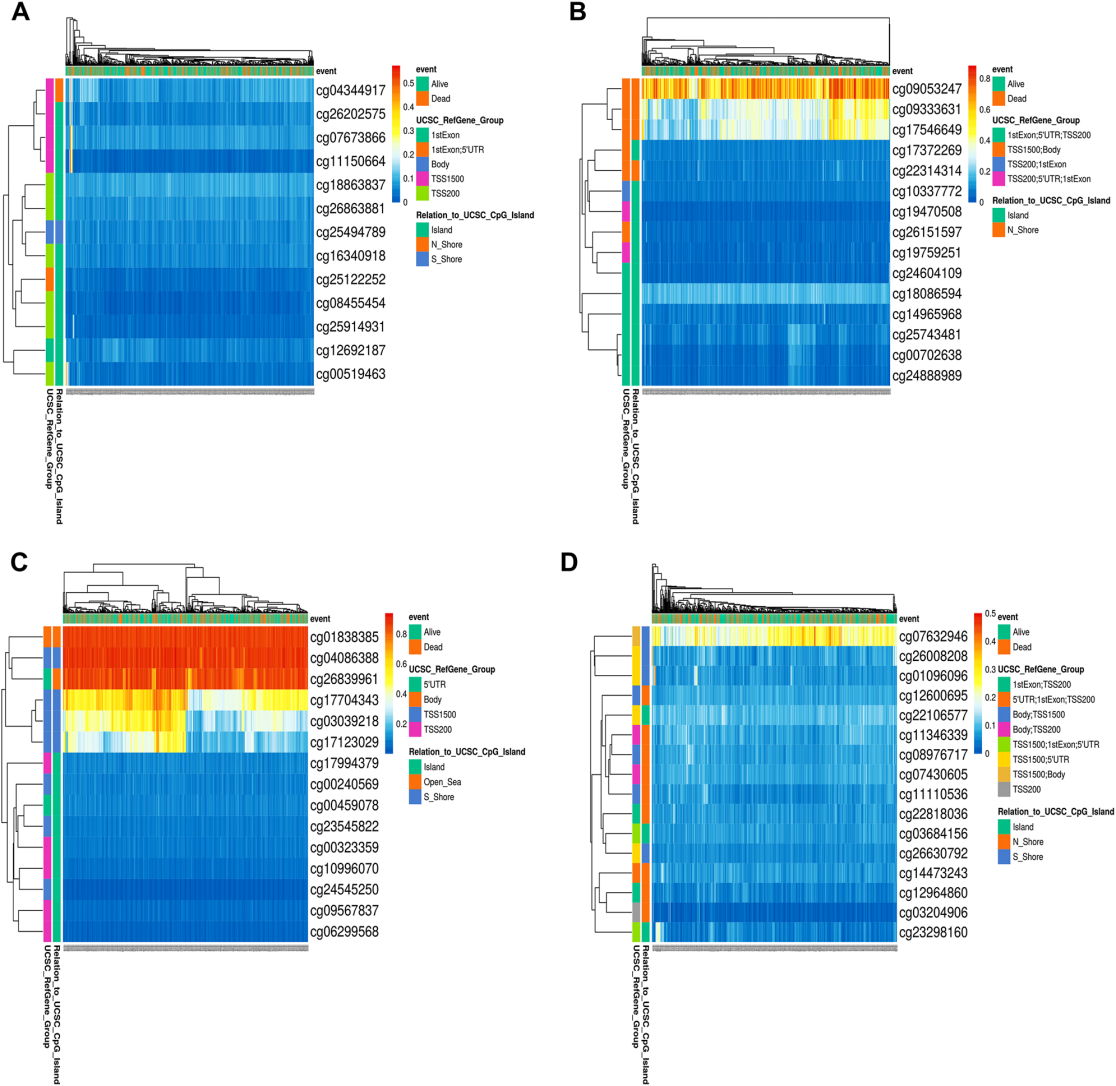

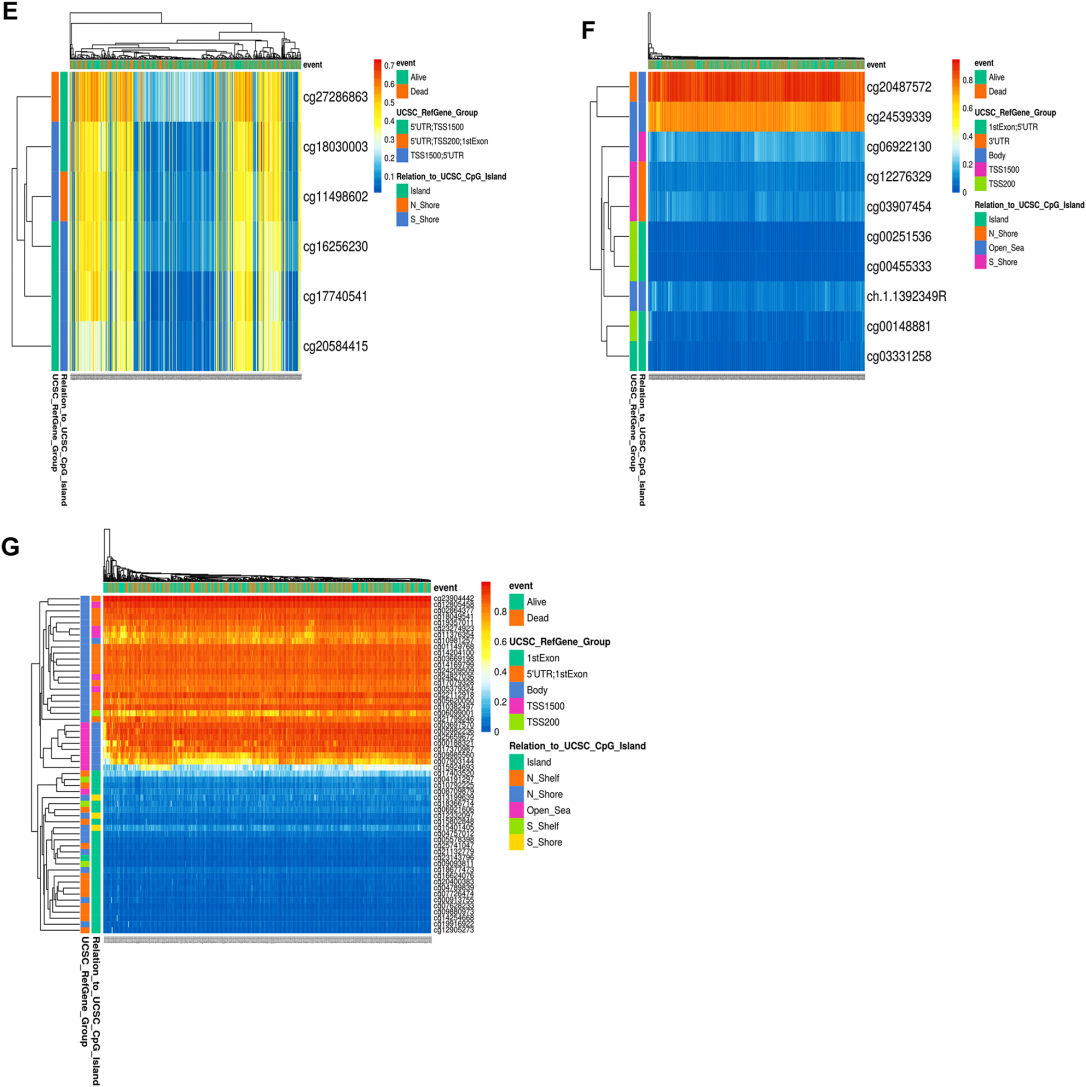
Heatmaps of CpG methylation levels of (**A**) KIF11, (**B**) KIF15, (**C**) KIF18B, (**D**) KIF20A, (**E**) KIF4A, (**F**) KIF2C, (**G**) KIFC1 across LUAD patients. Rows indicates the CpGs and columns indicates the patients. Methylation levels (1 = fully methylated; 0 = fully unmethylated) are shown as a continuous variable from red to blue color, high expression to low expression. Various colorful side boxes were used to represent the event, relation to UCSC_CpG_island and UCSC_refGene_Group.

**Figure 9:**
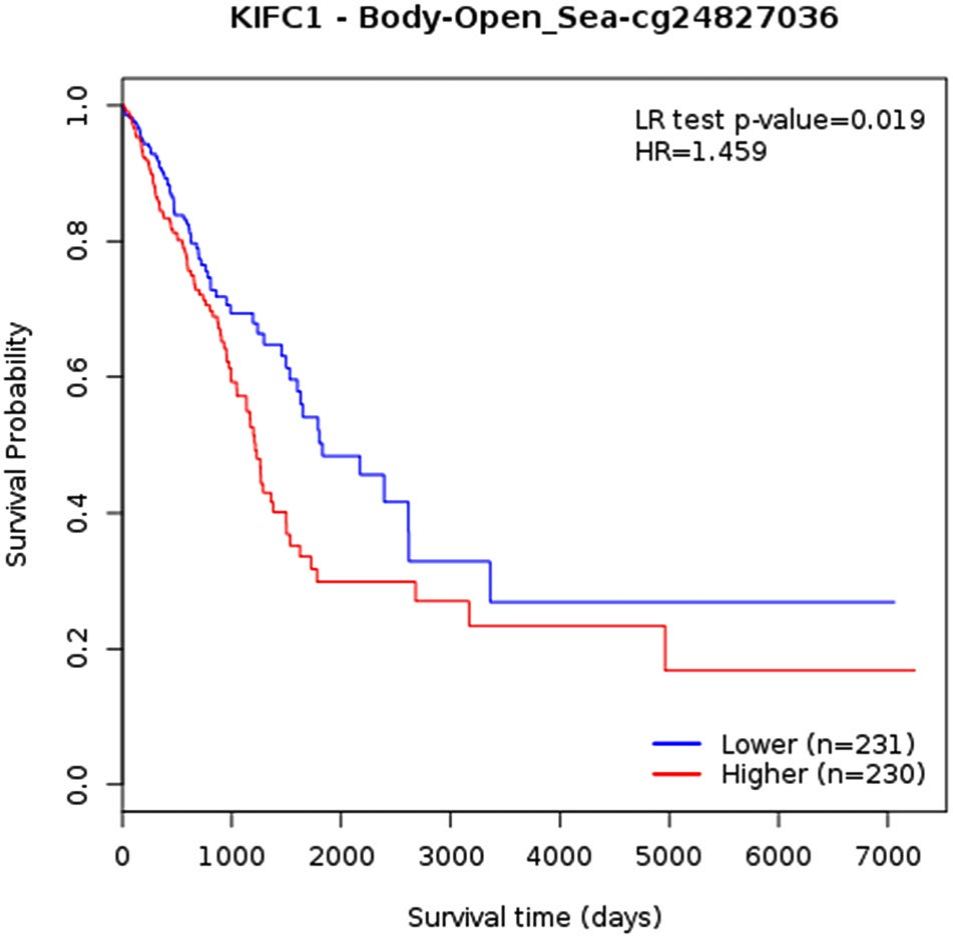
KM plot showing single CpG methylation of KIFC1 across LUAD patients. It’s location relative to CpG island, gene sub-region, CpG ID, and gene ID are also shown. Red and blue colors signify higher and lower expression groups.

We obtained the heatmaps of DNA methylation of selected kinesins using MethSurv. Among which cg04344917 CpG island (CGI) of *KIF11*, cg09053247 CGI of *KIF15*, cg01838385 CGI of *KIF18B*, cg07632946 CGI of *KIF20A*, cg20487572 CGI of *KIF2C*, cg27286863 CGI of *KIF4A*, cg2390442 CGI of *KIFC1* showed the highest methylation levels (Fig. 8). Furthermore, we studied KM plots which revealed that cg24827036 CGI of *KIFC1* were significantly associated with survival of LUAD patients (Fig. 9). A total of 461 patients were split into higher and lower expression groups. Higher methylated expression of *KIFC1* worsened the OS of LUAD patients.

## Discussion

LUAD’s malignancy results in high morbidity and fatality rate^35,36^. Despite advances in surgery, radiation, and chemotherapy, which have improved tumor patients' clinical prognosis and survival^37^, LUAD is still hard to treat because scientists don't fully understand the molecular mechanisms and basic signaling pathways in how LC works. It is expected that molecule-targeted therapy will be a revolutionary treatment technique for solid tumors, however, its efficacy and advantages remain restricted^38,39^. Because of chemoresistance and recurrence, the currently available therapeutic choices are limited. Therefore, a new and effective molecular target must be identified to cure LUAD.

Members of the KIF gene family are mostly found in eukaryotic cells, namely microtubules. Experiments conducted in vitro have shown that the transport of proteins occurs in only one direction, along the microtubule's negative pole and in the direction of the positive pole. Therefore, the genes that make up the KIF family are responsible for controlling the movement of mass proteins both inside of cells and outside of cells. This control encompasses a variety of functions, such as moving organelles and vesicles that contain material and taking part in the process of cell mitosis^15,40,41^. There have been reports that several genes in the kinesin family are linked to different kinds of cancer^42–44^. KIF family member genes have been demonstrated in various cancer types to establish their prognostic and diagnostic capacities. In our current study, we performed expression analysis of kinesin family in LUAD which revealed overexpression of *KIF11*/*12*/*15*/*23*/*18B*/*20A*/*2C*/*4A*/*C1* in tumor samples whereas *KIF17*/*26A*/*1C* were underexpressed in LUAD. Furthermore, we also studied mRNA expression based on cancer stage which showed overexpression of *KIF11*/*15*/*23*/*18B*/*20A*/*2C*/*4A*/*C1* in tumor tissues. Furthermore, we evaluated the prognostic value of selected kinesins in LUAD patients. Our results showed that an increased *KIF11*/*15*/*18B*/*20A*/*2C*/*4A*/*C1* expression is associated with poor OS in LUAD patients. So, by targeting these kinesins and decreasing their effect can be of therapeutic importance and patients’ survival can be increased.

Our findings corroborate with multiple previous findings that showed overexpression of *KIF11*, *KIF15*, *KIF18B*, *KIF20A*, *KIF2C*, and *KIF4A* in LUAD tissues and when LUAD patients have high expression of these KIFs, their chances of survival are lower^38,45–49^. Next, we studied the genomic alterations of key kinesins which showed the highest alteration in *KIFC1* (2.3%) as amplification being the most prominent type of alteration. Following that we constructed a PPIN of key kinesins and top 50 frequently altered genes and performed enrichment analysis. Our results showed high enrichment of kinesins in cell cycle and oocyte meiosis pathway, in biological processes named microtubule cytoskeleton organization involved in mitosis and mitotic spindle organization, microtubule binding and microtubule motor activity molecular functions and spindle and microtubule cytoskeleton cellular components.

For cells to divide and multiply, they go through a series of events known as the cell cycle, and abnormalities in the control of the genes involved in the cell cycle have been linked to the development of tumours. Mutations in upstream signal transduction pathways or genetic abnormalities within genes that encode cell cycle proteins cause cancer^50^. Our result showed high enrichment of kinesins in cell cycle processes hence kinesins are involved in controlling these processes somewhat and regulating LUAD.

The other two typically active mechanisms in LC were oocyte meiosis and progesterone-mediated oocyte maturation. One cycle of DNA replication in meiosis is followed by two cycles of chromosomal segregation (Meiosis I and Meiosis II). Normally, oocytes are stopped during the G2 stage of meiosis I. Progesterone exposure releases them from this natural lock, allowing the two meiotic division cycles to resume and the oocyte to mature^51,52^. So, it makes sense that dysregulations in oocyte maturation and meiosis would impact the cell cycle process, and further cell cycle changes would impact normal bodily functions, increasing the likelihood that one would develop cancer. So, cell cycle, progesterone-mediated oocyte maturation, and oocyte meiosis play prominent roles in the progression of LUAD^53^ and these two processes come under top 10 in the pathway enrichment analysis we did in our study showing the importance of kinesins in controlling these pathways in LUAD Microtubules are ${\upalpha }$- and ${\upbeta }$-tubulin heterodimers polymers. They exhibit highly dynamic behaviour, continuously engaging through processes of polymerization and de-polymerization, as well as lengthening and shortening. The fundamental components of the cytoskeleton are actin, intermediate filaments, and microtubules. They are crucial for various cell processes, including mitosis, the movement of vesicles and organelles inside cells, cell signaling, migration through cilia and flagella, cell shape and morphology^54^. So it can be said that any alteration in the function of kinesin from normal can impact these important pathways involved in LUAD progression.

We also performed tumor immune infiltration analysis on our key kinesins as tumor-infiltrating immune cells are critical components of the tumour microenvironment (TME), influencing tumor growth and survival depending upon their type and interaction LC clearly displays an invasion of a wide variety of immune cell types comprising neutrophils, natural killer (NK) cells, macrophages, dendritic cells, T cells & B cells^55^. These cells perform multiple purposes and combine or oppose one another, producing the LC TME. Neutrophils make up 50–70% of all white blood cells in the bloodstream and serve as the body's initial defence against infections. Neutrophils have been found to enhance tumor growth through various clinically relevant mechanisms. Tumor growth, angiogenesis, tumor cell migration, and metastasis are all facilitated by neutrophils. Still, a subclass of TANs known as N1 can have anticancer properties^56^. Our data revealed the highest correlation of our key kinesins with infiltration abundances of neutrophils in LUAD patients. CD8^+^T cell, also known as cytotoxic T lymphocytes, play a crucial role in mounting an efficient antitumor response. These cells can identify specific tumor-associated antigens (TAA) that are presented on major histocompatibility complex (MHC) class I molecules on the surface of cancer cells. Furthermore, they possess the ability to eliminate cancer cells directly^57^. Our study found that infiltrations of CD8^+^T cell and neutrophil correlated most with *KIF15* expression levels. Also, B cell and macrophage infiltrations correlated most with *KIF11* and *KIF4A* expression levels.

Cancer is often caused by the inactivation of several tumor-suppressor genes through point mutations and deletion of chromosomes^58^. Recent research has shown that epigenetic changes are key to cancer development. Many genes have CGIs in their promoter regions, and abnormal methylation of these sites in cancer leads to transcriptional suppression. Epigenetic alterations are passed down through cell division, resulting in gene activity change but no changes in the sequence of DNA^59,60^. Changes in DNA methylation patterns are a key feature of many types of cancer, including LC.

So further we conducted a single CpG methylation-based prognostic analysis on key kinesins which showed that CpG methylation in *KIFC1* was associated with poor prognosis in LUAD patients. *KIFC1* is believed to be an oncogene in various types of cancers as it plays a crucial role in clustering multiple centrosomes to sustain tumor survival^61,62^.

## Conclusions

Our research revealed a significant function for the kinesin family in initiating and progressing LUAD. KIF11/15/18B/20A/2C/4A/C1 mRNA expression levels were significantly upregulated and correlated with poor OS across LUAD patients. They were highly associated with cell cycle. Our results revealed the highest genomic alteration in *KIFC1* with highest number of CpG methylation. cg24827036 CGI of *KIFC1* was associated with poor prognosis across LUAD. We concluded that *KIFC1* can be a great individual prognostic biomarker, and that inhibiting its expression could be a potential therapeutic approach. Additionally, CpG island cg24827036 can serve as a great prognostic biomarker and treatment site.

## Supplementary Information

Supplementary Information 1.

Supplementary Table S2.

Supplementary Table S3.

## Data Availability

The data that support the findings of this study are available from the corresponding author upon reasonable request.
