# Supplementary material for: Investigating the role of Kinesin family in lung adenocarcinoma via integrated bioinformatics approach
Source: Sci Rep. 2023 Jun 17;13:9859. doi: 10.1038/s41598-023-36842-6 (PMC10276827; doi:10.1038/s41598-023-36842-6)
Supplement: Supplementary file 1 — Supplementary Information 1. [file 41598_2023_36842_MOESM1_ESM.docx]

**Supplementary Information**

**Investigating the role of Kinesin family in lung adenocarcinoma via integrated bioinformatics approach**

Gulnaz Tabassum**^1,†^**, Prithvi Singh**^2,†^**, Rishabh Gurung**^2^**, Mohammed Ageeli Hakami**^3^**, Nada Alkhorayef**^3^**, Ahad Amer Alsaiari**^4^**, Leena S. Alqahtani**^5^**, Mohammad Raghibul Hasan**^3^**, Summya Rashid**^6^**, Atul Kumar**^1^**, Kapil Dev**^1,*^**, Ravins Dohare**^2,*^**

**^1^**Department of Biotechnology, Faculty of Natural Sciences, Jamia Millia Islamia, New Delhi 110025, India

**^2^**Centre for Interdisciplinary Research in Basic Sciences, Jamia Millia Islamia, New Delhi 110025, India

**^3^**Department of Clinical Laboratory Sciences, College of Applied Medical Sciences, Al- Quwayiyah, Shaqra University, Riyadh 13343, Saudi Arabia

**^4^**Department of Clinical Laboratory Sciences, College of Applied Medical Sciences, Taif University, Taif 21944, Saudi Arabia

**^5^**Department of Biochemistry, College of Science, University of Jeddah, Jeddah 23445, Saudi Arabia

**^6^**Department of Pharmacology & Toxicology, College of Pharmacy, Prince Sattam Bin Abdulaziz University, Alkharj 16278, Saudi Arabia

**^†^**These authors contributed equally to this work as co-first authors.

**^*^Correspondence:** Dr. Ravins Dohare and Dr. Kapil Dev

**Table S1.** *Hazard ratio (HR) with 95% confidence interval (CI), logrank p-value, median survival time of KIF11, KIF15, KIF18B, KIF20A, KIF2C, KIF4A, KIFC1 in lower and higher expression microarray LUAD cohort.*

| **Gene symbol** | **Low expression cohort (months)** | **High expression cohort (months)** | **Hazard Ratio (HR) with 95% Confidence interval (CI)** | $\mathbf{logrank p-value}$ |
| --- | --- | --- | --- | --- |
| ***KIF11*** | $92.97$ | $63.4$ | $1.26 (1.05 - 1.5)$ | $0.011$ |
| ***KIF15*** | $99$ | $57$ | $1.53 (1.29 - 1.82)$ | ${8.5\times10}^{-7}$ |
| ***KIF18B*** | $96.2$ | $49$ | $1.62 (1.37 - 1.93)$ | ${2.5\times10}^{-8}$ |
| ***KIF20A*** | $103$ | $49.97$ | $1.67 (1.41 - 1.98)$ | ${2.9\times10}^{-9}$ |
| ***KIF2C*** | $107$ | $48$ | $1.9 (1.6 - 2.25)$ | ${1.0\times10}^{-13}$ |
| ***KIF4A*** | $96.2$ | $46.7$ | $1.71 (1.44 - 2.03)$ | ${8.7\times10}^{-10}$ |
| ***KIFC1*** | $107$ | $49.97$ | $1.77 (1.49 - 2.1)$ | ${4\times10}^{-11}$ |


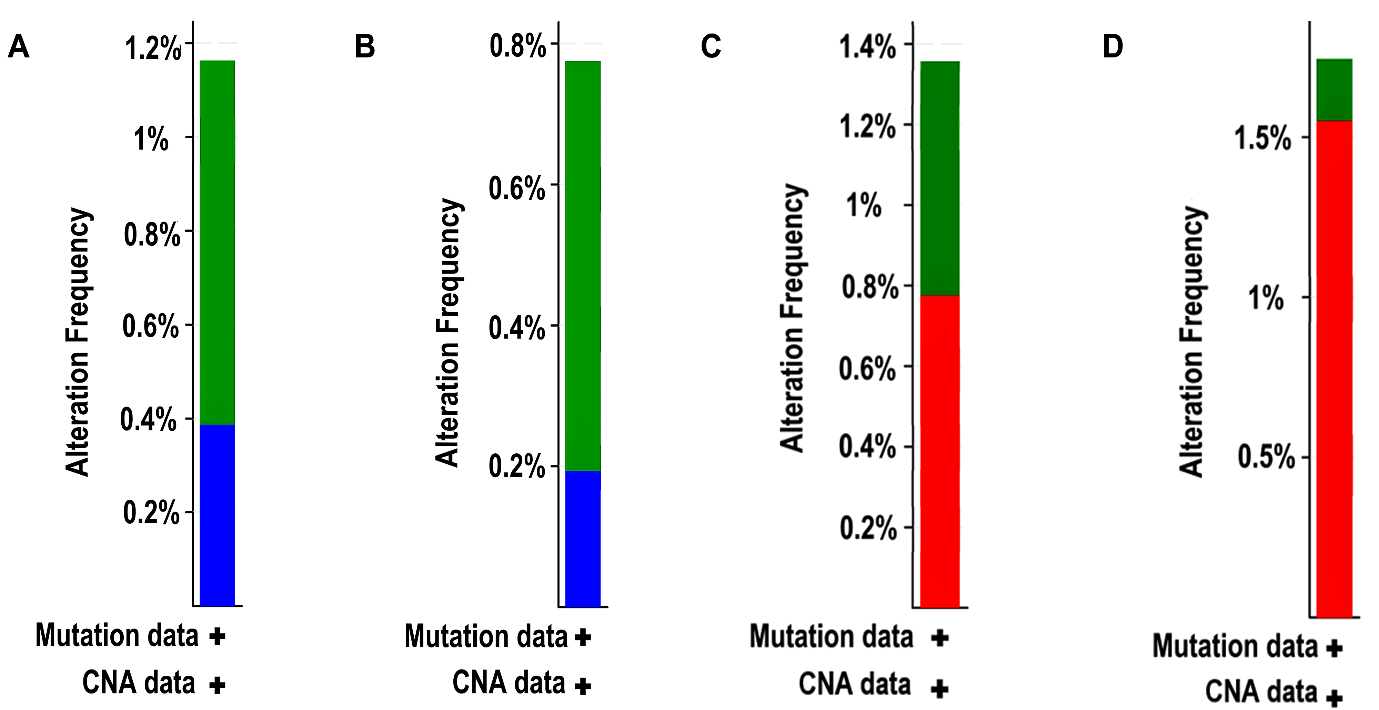


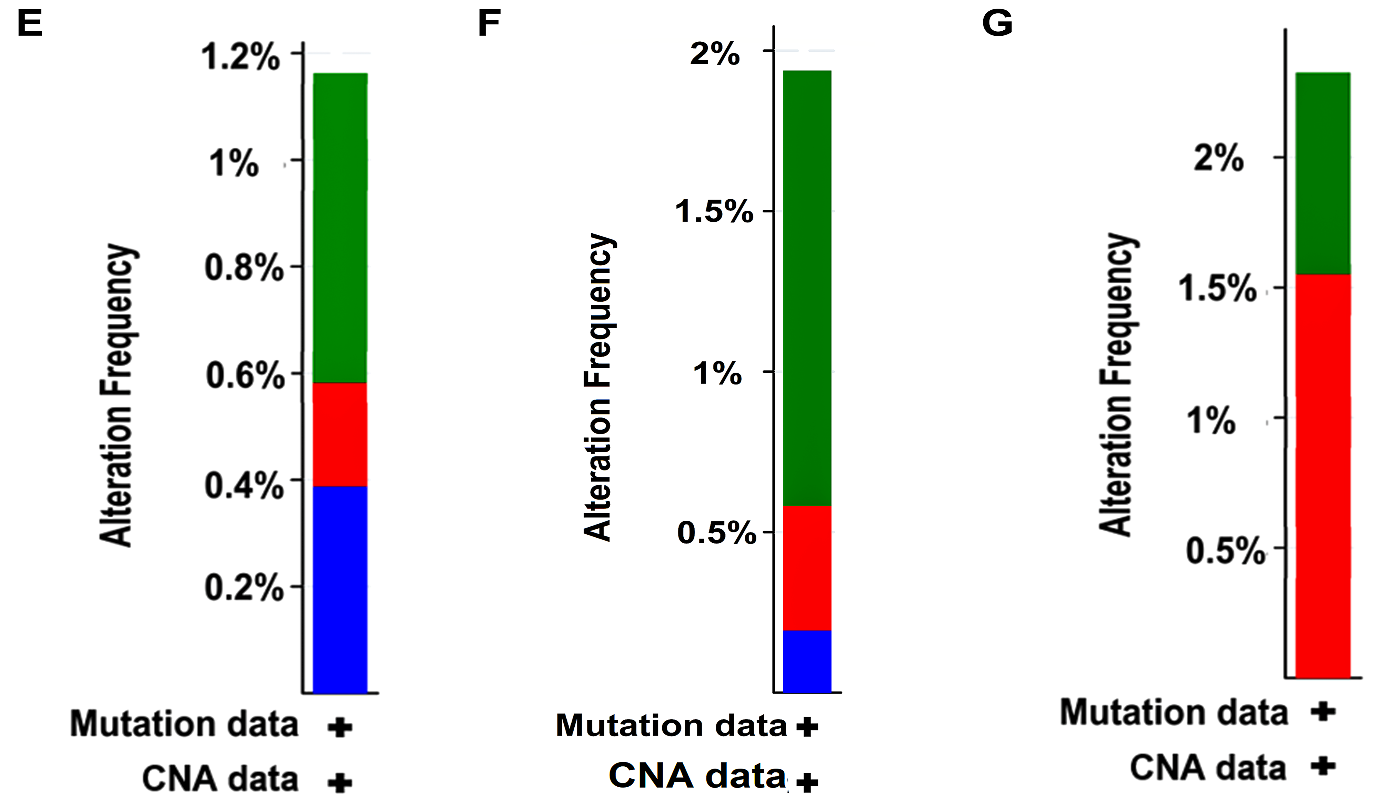


**Figure S1.** *Barplots showing alteration frequencies of* ***(A)*** *KIF11,* ***(B)*** *KIF15,* ***(C)*** *KIF18B,* ***(D)*** *KIF2C,* ***(E)*** *KIF20A,* ***(F)*** *KIF4A, (G) KIFC1 across TCGA-LUAD cohort. Red, blue, and green colored bars signify amplifications, deep deletions, and missense mutations.*


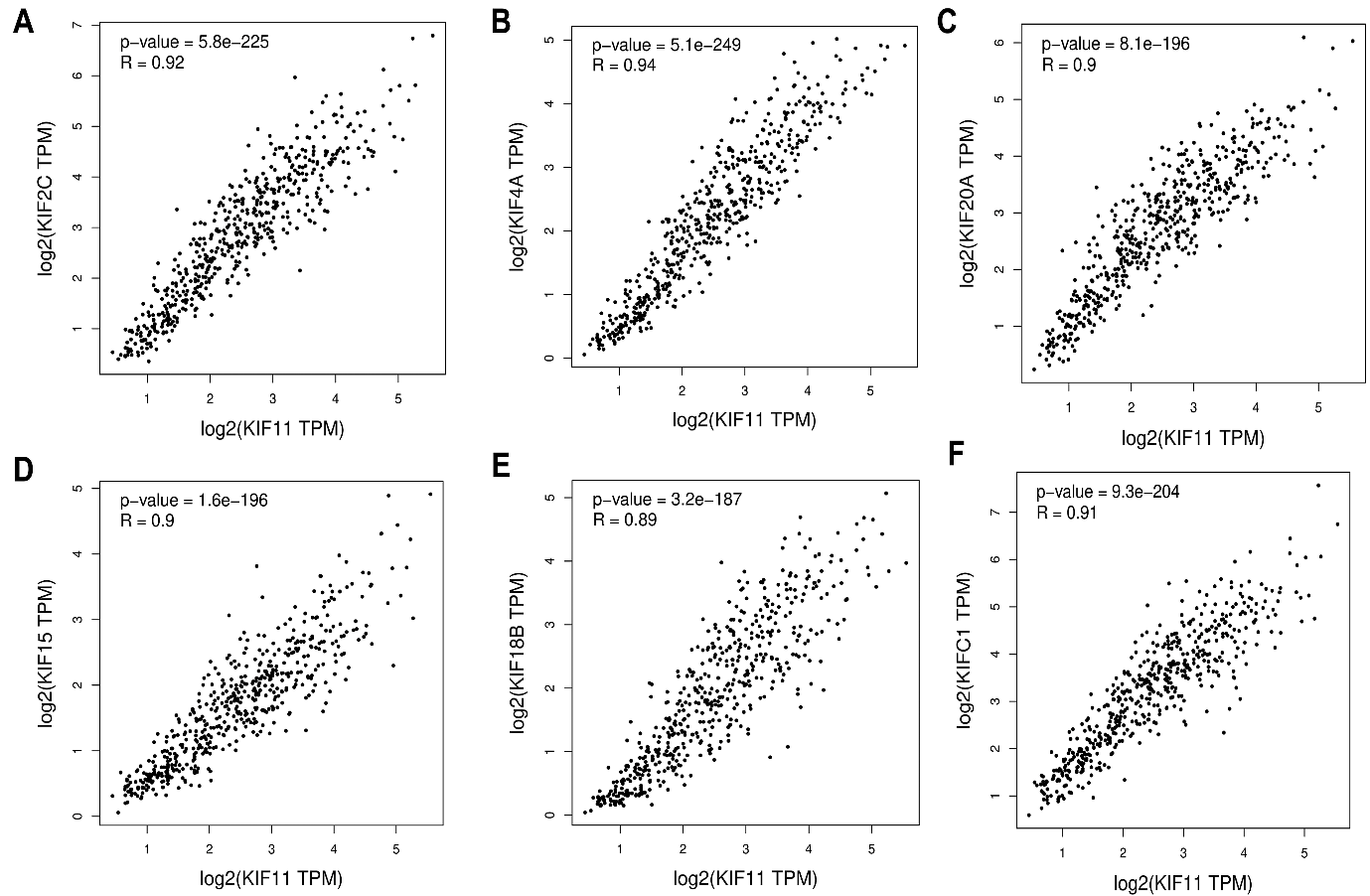


**Figure S2.** *Scatterplots showing spearman pairwise correlations between* ***(A)*** *KIF11 and KIF2C,* ***(B)*** *KIF11 and KIF4A,* ***(C)*** *KIF11 and KIF20A,* ***(D)*** *KIF11 and KIF15,* ***(E)*** *KIF11 and KIF18B, (F) KIF11 and KIFC1.*


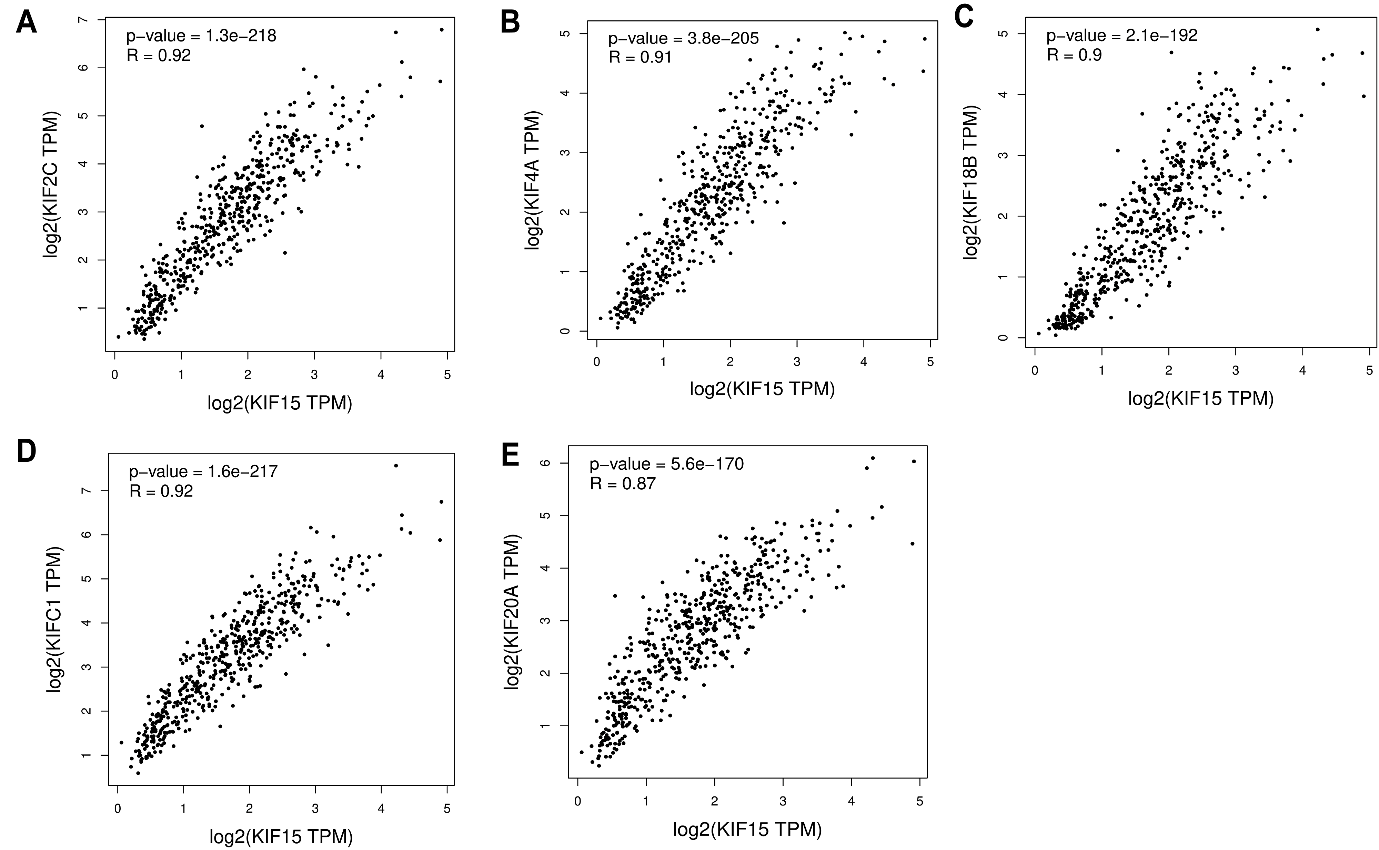


**Figure S3.** *Scatterplots showing spearman pairwise correlations between* ***(A)*** *KIF15 and KIF2C,* ***(B)*** *KIF15 and KIF4A,* ***(C)*** *KIF15 and KIF18B,* ***(D)*** *KIF15 and KIFC1,* ***(E)*** *KIF15 and KIF20A.*


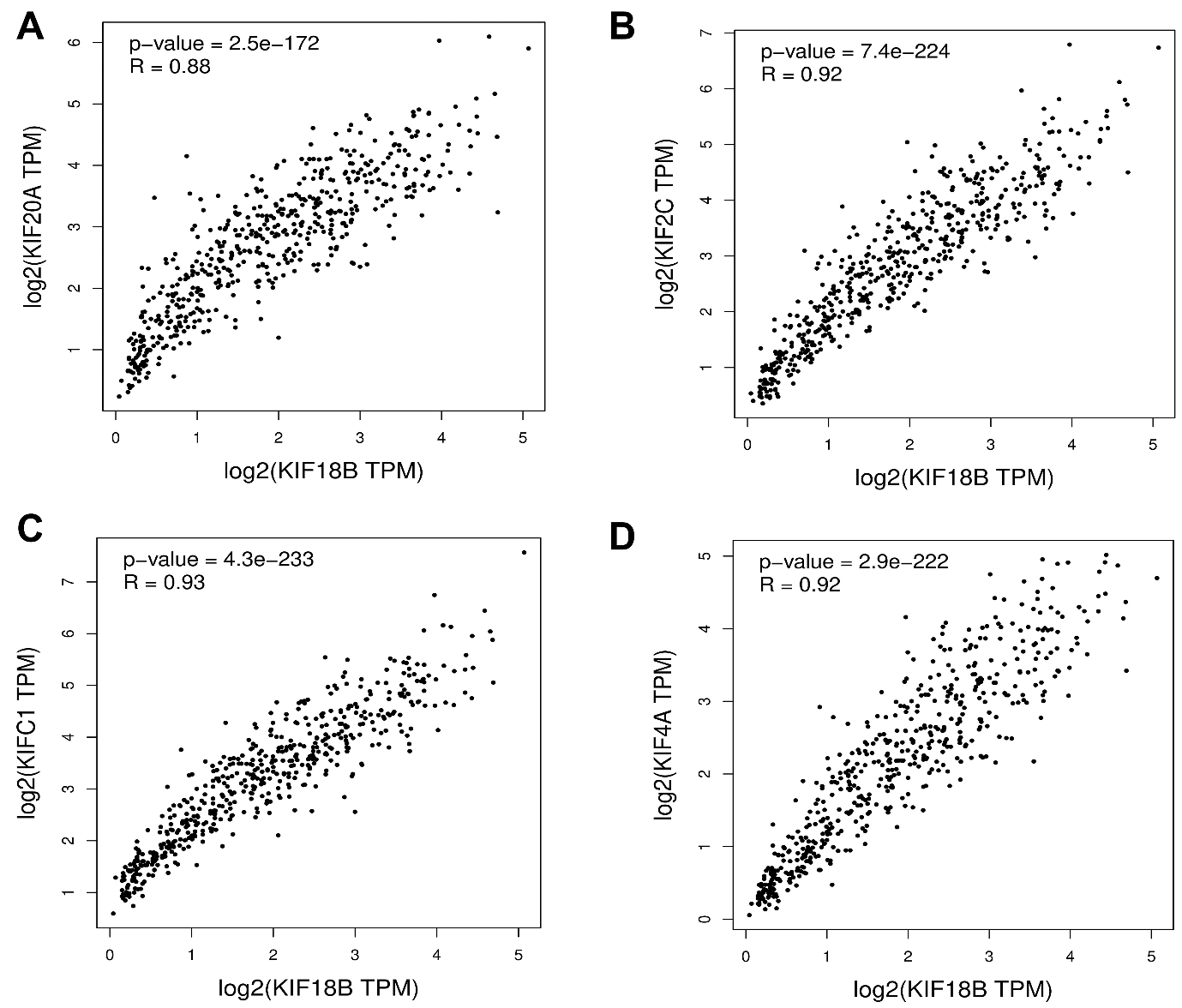


**Figure S4.** *Scatterplots showing spearman pairwise correlations between* ***(A)*** *KIF18B and KIF20A,* ***(B)*** *KIF18B and KIF2C,* ***(C)*** *KIF18B and KIFC1,* ***(D)*** *KIF18B and KIF4A.*


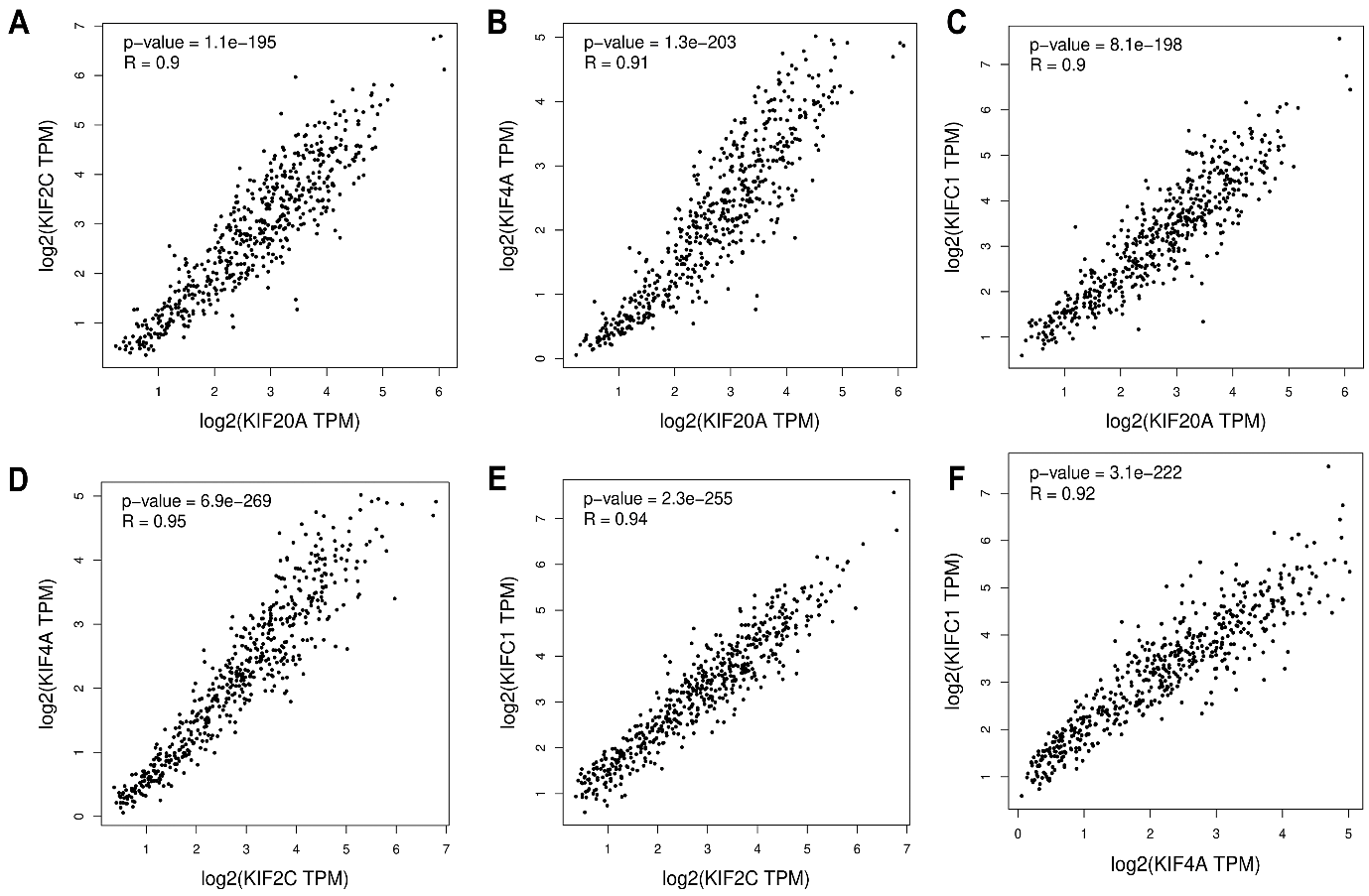


**Figure S5.** *Scatterplots showing spearman pairwise correlations between* ***(A)*** *KIF20A and KIF2C,* ***(B)*** *KIF20A and KIF4A,* ***(C)*** *KIF20A and KIFC1,* ***(D)*** *KIF2C and KIF4A,* ***(E)*** *KIF2C and KIFC1, (F) KIF4A and KIFC1.*


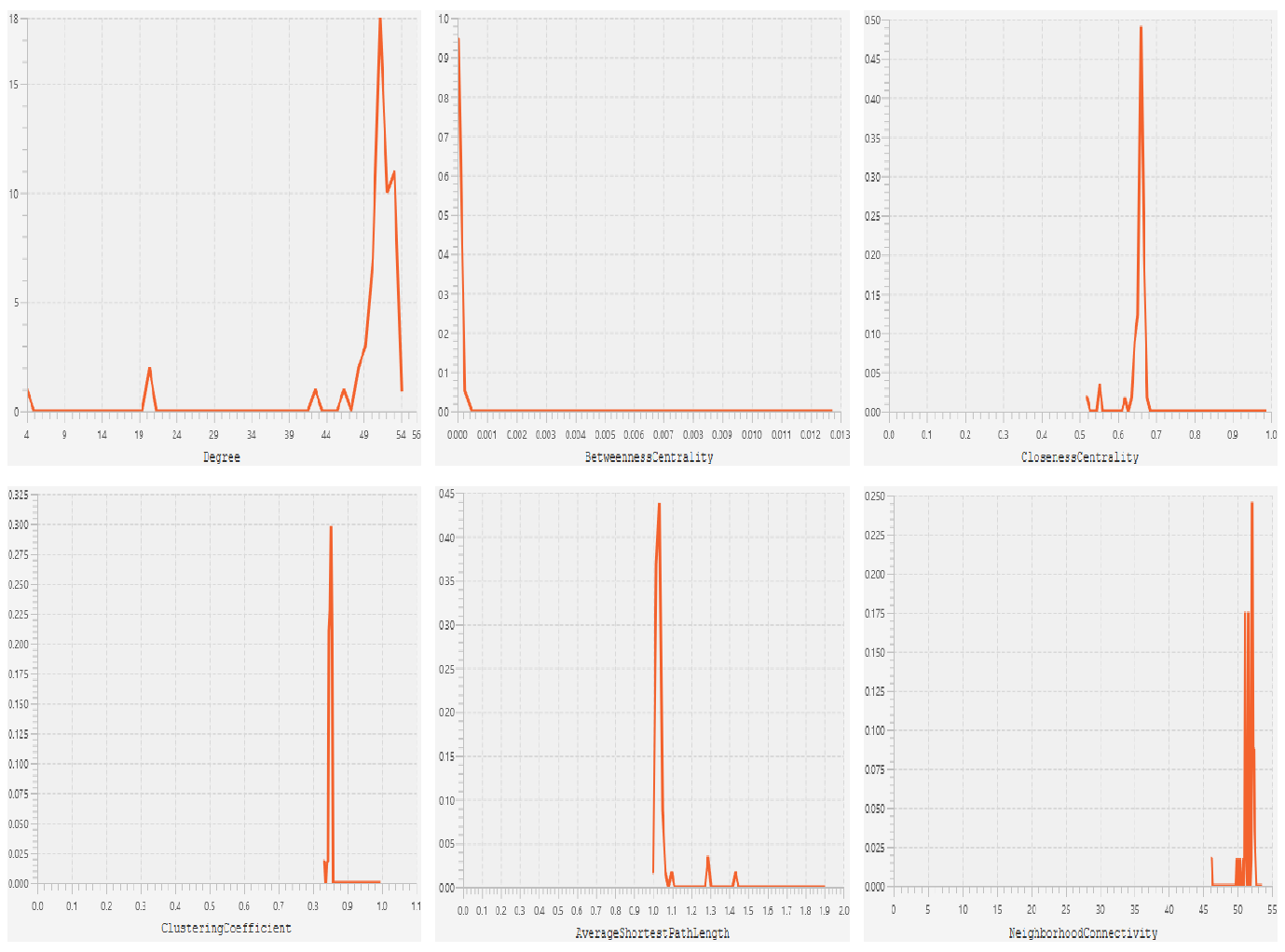


**Figure S6.** *Centrality measures showing node degree distribution, betweenness, closeness, clustering coefficient, average shortest path length, neighborhood connectivity of PPIN.*
